# Recent advances in bioreactors for cell-based therapies

**DOI:** 10.12688/f1000research.12533.1

**Published:** 2018-04-30

**Authors:** Makeda Stephenson, Warren Grayson

**Affiliations:** 1 Translational Tissue Engineering Center, Johns Hopkins University School of Medicine, Baltimore, Maryland, USA; 2 Department of Biomedical Engineering, Johns Hopkins University School of Medicine, Baltimore, Maryland, USA; 3 Department of Materials Science and Engineering, Johns Hopkins University, Baltimore, Maryland, USA; 4 Institute for NanoBioTechnology, Johns Hopkins University, Baltimore, Maryland, USA

**Keywords:** Bioreactors, stem cell manufacturing, tissue-on-a-chip

## Abstract

Bioreactors have become indispensable tools in the cell-based therapy industry. Various forms of bioreactors are used to maintain well-controlled microenvironments to regulate cell growth, differentiation, and tissue development. They are essential for providing standardized, reproducible cell-based products for regenerative medicine applications or to establish physiologically relevant *in vitro* models for testing of pharmacologic agents. In this review, we discuss three main classes of bioreactors: cell expansion bioreactors, tissue engineering bioreactors, and lab-on-a-chip systems. We briefly examine the factors driving concerted research endeavors in each of these areas and describe the major advancements that have been reported in the last three years. Emerging issues that impact the commercialization and clinical use of bioreactors include (i) the need to scale up to greater cell quantities and larger graft sizes, (ii) simplification of *in vivo* systems to function without exogenous stem cells or growth factors or both, and (iii) increased control in the manufacture and monitoring of miniaturized systems to better capture complex tissue and organ physiology.

## Introduction

Bioreactors provide controlled delivery of nutrients and biomimetic stimuli in order to influence cell growth, differentiation, and tissue formation. They have been extensively used to promote the expansion of red blood cells, chimeric antigen receptor (CAR) T cells, induced pluripotent stem cells, and mesenchymal stem cells. Additionally, the ability to control the spatiotemporal delivery of the biological, biochemical, and biophysical signals that regulate tissue development confers a number of advantages for engineering 3D tissues relative to standard cell culture techniques by providing well-defined conditions to regulate cell behaviors. These advantages include (i) improved standardization and reproducibility, (ii) scale-up to larger, clinically relevant tissue grafts or cell expansion scales, (iii) superior functionality compared with 3D grafts cultured in tissue culture flasks, and (iv) improved systems for testing cell responses to a range of experimental parameters. As the field of regenerative medicine has matured, the number of applications has increased and the roles that bioreactors play in enabling the commercialization and clinical translation of stem cell-based technologies have become more defined. In this review, we will provide a critical overview of biomedical applications of bioreactors and discuss current trends and recent advances that promote the application of bioreactor technologies for single-cell manufacture, production of engineered tissue grafts, and drug screening.

## Bioreactors for cell proliferation and differentiation

The therapeutic promise of stem cell-based technologies for the treatment of pathologies ranging from hair loss ^1^ to blindness ^2^ has precipitated the need for a cell-manufacturing sector to provide therapeutic allogeneic cells. Owing to the extensive infrastructure requirements and rigorous standards defined by regulatory agencies, the cost will likely be too burdensome for traditional hospitals and treatment centers and will manifest as centralized facilities that specialize in providing high-quality cells with verifiable characteristics. However, cell-based therapies often require the application of vast quantities of cells (10 ^8^–10 ^10^) in order to be effective. A practical limitation arises as the amount of space required to grow these large quantities of cells using standard cell culture apparatus is prohibitive. This has spawned a demand for bioreactors capable of supporting industrial-scale, ultra-high-density cell suspension cultures with controlled microenvironments, standardization, and uniformity of culture conditions in order to generate homogenous populations of stem or lineage-specific cells. A few types of bioreactors have been employed to generate large populations of phenotypically defined cells. Variable designs have been employed for adherent versus non-adherent cells and to account for differences in cellular responses to microenvironmental cues. Table 1 summarizes several bioreactor types that have been used for cell expansion.

**Table 1.** Bioreactor systems for cell expansion.

Bioreactor type	Commercial examples	Parameter ranges	Advantages/limitations	Example case studies	Reference
Rocking bed (wave motion)	• WAVE (GE Healthcare) • Finesse (Thermo Fisher) • Biostat (Sartorius)	• Size (1–500 L) • Rocking angle: 5–35° • Rotation speed: 10–35 rpm	Advantages: • Versatile single-use bags Limitations: • Limited scale-up potential	• Cell type: hMSCs • Method:microcarrier culture • Culture time: 7 days • Fold expansion: 0.7–14.5 • Metrics: viability, tri-lineage differentiation, aggregate size	3
Stirred tank	• Mobius (EMD Millipore) • Finesse (Thermo Fisher)	• Size (100 mL–1,000 L) • Impeller power/speed: variable during culture period • Impeller design: updraft or downdraft, single or multiple	Advantages: • Functional at-large volumes: >50 L Limitations: • Shear forces may impact cell viability/differentiation	• Cell type: hMSCs, hASCs, hiPSCs, and murine ovary cell cells • Method: aggregates, microcarriers, and single-cell suspensions • Culture time: 11–17 days • Fold expansion: 25.7–43 • Metrics: viability, aggregate size, and differentiation capacity	6, 7, 11, 17
Rotating wall vessels	RCCMAX (Synthecon)	• Size (100 mL–10 L) • Rotational speed: 5–20 rpm • Continuous medium recirculation or closed batch system	Advantages: • Low turbulence • Can simulate microgravity Limitations: • Effective only at small volumes: <10 L	• Cell type: hMSCs • Method: scaffolds • Culture time: 21 days • Fold expansion: ~39 • Metrics: viability, surface marker expression, and differentiation	18
Perfusion bioreactor	• FiberCell (FiberCell Systems) • Quantum Cell Expansion (Terumo BCT)	• Size (100 mL–5 L) • Perfusion: direct (for example, through scaffolds) or indirect (hollow-fiber, encapsulated cells)	Advantages: • Limited turbulence • Can be automated • Compact Limitations: • Shear forces may impact cell viability/differentiation	• Cell type: hMSCs • Method: encapsulation • Culture time: 21 days • Fold expansion: not applicable • Metrics: viability and differentiation	19, 20
Isolation/expansion automated systems	• G-Rex (Wilson Wolf) • CliniMACs Prodigy (Miltenyi Biotec)	• Size (100 mL) • Degree of automation	Advantages: • Versatile single-use bags • Automated cell isolation, manipulation, and expansion • GMP-compliant Limitations: • Primarily T-cell expansion	• Cell type: human lymphocytes • Method: suspension culture • Culture time: 8–14 days • Fold expansion: 32–63 • Metrics: viability and cell marker evaluation	21, 22

GMP, good manufacturing practices; hASC, human adipose-derived stem cell; hiPSC, human induced pluripotent stem cell; hMSC, human mesenchymal stem cell.

### Adhesion-dependent cell types

Since many therapeutically relevant cells are adhesion dependent and thus cannot be readily grown in suspension cultures, the scale-up of cell manufacture presents a unique challenge. To overcome this obstacle, biomaterial technologies have been combined with bioreactors to support the development of high-density bioreactor conditions. For adherent cells, suspension culture can be achieved by the use of hollow fibers in perfusion systems, encapsulation, or microspheres (also known as microcarriers), which increase the surface area of a suspension bioreactor ^3^. Packed bed bioreactors have also been used to enable both isolation and expansion of mesenchymal stem cells ^4, 5^. Specifically, studies have shown that adherent cells such as bone marrow-derived mesenchymal stem cells can be cultured on protein-coated microspheres. Cells grown in this manner can retain their functional markers and viability ^3^. With this strategy, it is possible to scale the volumes of cell cultures up to the order of 10 ^2^–10 ^3^ L and stand-alone systems such as the Mobius (EMD Millipore) stirred-tank bioreactor series are commercially available in sizes ranging from 50 to 2,000 L. At this scale, the impeller speeds required to maintain homogenous distribution of metabolites generate turbulent flows and large shear forces, which induce spontaneous differentiation of stem cells. In order to mitigate this effect, studies have focused on either optimizing agitation schemes ^6, 7^ or encapsulating cells in microspheres ^8, 9^. Although these strategies are promising for providing commercially available therapeutic cells, the high cost of reagents and growth factors restricts the use of industrial-scale systems in scientific exploration ^10^.

Published studies of bioreactor cultures of mesenchymal- and adipose-derived stem cells typically report data for bioreactors with maximum volumes of 3 L ^11, 12^, although Lawson *et al*. demonstrated the ability of these systems to safely and effectively enable a 43-fold cell expansion over 11 days using a 50 L volume bioreactor with a graduated agitation and feeding scheme ^11^. The cells retained their tri-lineage pluripotency, T-cell modulation behavior, and phenotypic markers, including CD44 and CD90, when compared with cells cultured under traditional conditions ^11^. However, these studies used growth factors and animal serum in their media. The development of defined media without supraphysiological concentrations of growth factors in cell culture would promote economically feasible industrial-scale culture. Another key shortcoming is the dearth of *in vivo* efficacy data from cells produced in these large-scale bioreactors.

Another strategy for enhancing the therapeutic effectiveness of mesenchymal- and adipose-derived stem cells has been to deliver them as self-assembled aggregates. These cellular spheroids exhibit enhanced survival and tissue-forming properties ^13– 15^. The impact of bioreactors on mesenchymal stem cell aggregation kinetics and spheroid size has recently been studied by using commercially available WAVE Bioreactors™, which provide gentle stirring and single-use bags for scale-up ^16^. The authors used a combination of experimentation and modeling to demonstrate that a tightly controlled size distribution of cellular aggregates with enhanced therapeutic characteristics could be obtained.

### Induced pluripotent stem cell expansion

Suspension aggregate cultures in rotating flasks, rotating wall bioreactors, stirred-tank bioreactors, and WAVE Bioreactors™ have become the primary means for the expansion of embryonic stem cells or induced pluripotent stem cells (reviewed in 23, 24). Aggregate cultures are thought to more closely mimic the native microenvironment (inner cell mass) of pluripotent cells. Owing to their high differentiation capacity, the microenvironmental conditions for pluripotent cells—including aggregate size—must be tightly regulated in order to maintain an undifferentiated phenotype. The aggregate sizes are regulated by chemical (Rho-kinase [ROCK] inhibitors) and mechanical (shear forces or physical disruption) techniques. In addition, the dissolved oxygen concentrations and dilution rate impact the differentiated state of the cells ^25^. Using this continuous expansion method in a stirred-tank bioreactor, Abecasis *et al*. demonstrated 1,100-fold expansion in 11 days using 4% dissolved oxygen ^25^. The resulting cells were characterized by using proteomic and gene stability analysis as well as proliferation and gene expression assays to validate their naïve state ^25^.

### CAR T-cell expansion

The use of CAR T cells and other immune cells such as natural killer cells is an important emerging therapy for treating diseases of the immune system and is being applied to patient-specific treatment of cancer ^26^. CAR T-cell therapy uses autologous T cells expanded to therapeutic volumes (liter scale) in rocking bed, disposable bag bioreactors such as WAVE™ or CultiBag bioreactors. Technologies for the automated or semi-automated processing of CAR T cells are commercially available and include the CliniMACS Prodigy, DynaMag, and G-Rex systems ^27^.

Recent published studies demonstrate the ability of isolation and transduction protocols to be scaled up to clinical production in ways that comply with good manufacturing practices and clinical regulatory standards. These studies focused on optimizing production processes and verifying and characterizing the products using the automated CliniMACS Prodigy system ^21, 22^.

### Quality criteria for cell-based products

Finally, cell products from bioreactors must be evaluated in a standardized manner to ensure quality control. The International Society for Cell Therapy and the European Society for Blood and Marrow Transplantation publish joint quality guidelines for identifying cell products ^28, 29^. These may include key release criteria such as surface marker analysis, proteomics, functional assays, and sterility testing.

## Advances in tissue engineering bioreactors

In contrast to bioreactors that produce single cells, tissue engineering bioreactors have supported the development of large 3D tissue grafts. To produce large (centimeter-sized) viable grafts, these systems often use convective flow to provide crucial mass transport regimes, overcome the diffusional limitations of nutrients and oxygen, and prevent the accumulation of metabolic waste products that otherwise induce starvation and death of the cells in the inner regions of the construct. Tissue engineering bioreactors can also enhance the functionality of grafts through the application of biomimetic physiological stimuli as well as the incorporation of sensors that give real-time feedback of culture conditions. After incubation, the mature, functional cellular constructs can be transplanted *in vivo* to regenerate damaged tissues. Tissue engineering bioreactors will likely play a significant role in translating engineered grafts to the clinic as the potential automation renders them economically efficient and amenable to mass production for larger populations of patients.

Cutting-edge research in this field continues to focus on the improved application of biophysical stimuli to optimize functional tissue assembly ^30– 35^ and computational modeling to improve predictability of the outcomes ^19, 36, 37– 40^. Additionally, notable efforts to enhance the clinical applicability of these grafts have focused on engineering grafts that are similar in size to critical-sized bone defects in humans and are tailored to the patient ^20, 41– 42^. Nguyen *et al*. recently demonstrated the ability to culture a 200 cm ^3^ cell-based construct *in vitro* without the development of necrotic centers ^20, 42^. In this approach, bone marrow-derived mesenchymal stem cells were encapsulated in hydrogel beads and placed in a tubular perfusion bioreactor. Three-dimensional-printed molds that could be anatomically shaped were used to direct the flow through the hydrogel beads. The space between the hydrogel beads enhanced mass transport to the cells throughout the entire construct, allowing the stem cells to remain viable and undergo osteogenic differentiation. Although this approach represents a critical advancement in the culture of clinically sized constructs, it remains limited by the use of hydrogel beads that minimize cell–cell interactions and inhibit paracrine signaling between cells, which are important factors in bone formation. In contrast, Bhumiratana *et al*. directly seeded adipose-derived stem cells into the pore spaces of anatomically shaped, porcine temporomandibular joint scaffolds ^41^. They cultured the adipose-derived stem cell-seeded scaffolds in perfusion bioreactors for 3 weeks *in vitro* before using the bioreactors to maintain their viability during transport to an on-site animal facility. The grafts—customized for each pig—were implanted and cultured for up to 6 months *in vivo*. This was a foundational, proof-of-concept study and clearly demonstrated the feasibility of using this strategy as a treatment for humans. However, in general, there remains a huge gap in the growth of 3D engineered tissues in bioreactors and demonstration of *in vivo* functional integration and potency.

## *In vivo* bioreactors

In spite of the inherent advantages of using tissue engineering bioreactors to grow entire grafts that are primed for implantation into defect sites, there are a number of practical barriers to clinical translation associated with extended *ex vivo* culture. One major limitation is that the large, volumetric grafts often lack an intact vasculature, which consequently hampers their post-transplantation viability. To overcome these limitations, an alternative approach known as “ *in vivo* bioreactors” has been employed. Unlike the systems described above, the *in vivo* bioreactor, despite the use of the “bioreactor” terminology, does not incorporate robust design principles or the development of new equipment. There is no hardware, and the success of the strategy is highly dependent on surgical expertise and manipulation. Rather, it primarily refers to a pocket within the body into which biomaterials or immature tissue engineered constructs are surgically implanted and incubated for an extended period of time. Within these pockets (for example, omentum or muscle flap), the grafts harness the regenerative capacity of the body to become fully vascularized. Key advantages of this method include the presence of naturally occurring cytokines and other factors, the establishment of neovasculature and nervous tissue within the implant, and immune compatibility ^43^. The primary application of the *in vivo* bioreactor principle has been for the development of critical-sized bone grafts. Several recent studies have demonstrated the use of prefabricated bone grafts which are either incubated *in situ* or vascularized by extended implantation in muscle or omentum or anastomosed with large arteries ^43– 53^ and may be feasible even without the use of transplanted stem cells or growth factors.

## Organ-on-a-chip bioreactors

As the previous examples illustrate, bioreactors typically have been employed to address challenges of scale-up. However, miniaturized tissues created by using microfluidic bioreactors facilitate efficient, inexpensive, high-throughput drug screening or disease modeling. Microfluidic bioreactors—often referred to as lab-on-a-chip systems—use minute quantities of cells grown together in micrometer-scaled wells. Microliter volumes of fluid are pumped to the cells through channels that allow the effects of multiple concentrations of growth factors or pharmacological agents to be rapidly tested. Often, modified cells are used to permit easily monitored parameters (such as fluorescence ^54^) to be used as read-outs of cellular responses. Early modifications to these systems enabled the use of high-density 3D cell culture using multi-cell aggregates, microspheres, and cell encapsulation to better recapitulate the cell–cell interactions of native tissues in ways not possible in 2D culture. Even so, it is challenging to replicate the impact of pharmacological agents on the complex functions of tissues, such as the lung or heart, in these simplified systems. Hence, more recent versions of lab-on-a-chip bioreactors have incorporated physiological factors such as airflow and mechanical stimulation that mimic breathing ^55, 56^ or have integrated vasculature and direct blood flow with contractile cardiac cells ^57^. These two technologies, which are currently being commercialized, more accurately capture physiological responses to specific stimuli while retaining the benefits of simplicity and low cost.

The most recent developments in the field of lab-on-a-chip technology have focused on increasing the ease of use. For example, researchers are investigating methods to 3D print and seed an entire chip in a single pass ^54, 58^. Other teams have developed smartphone-based systems to monitor the internal environment ^59^ and hybrid materials which allow point-by-point manipulation of the cells within the bioreactor ^60^. Perhaps one of the most significant advantages of lab-on-a-chip systems is their ability to capture complex physiology of multiple organ systems. Lee *et al*. ^61^ and Shirure and George ^62^ reported on the development of pumpless, dual-organ bioreactor systems. Current trends portend the advent of more advanced human-on-a-chip systems, which will test on- and off-target effects of drugs on multiple organ systems.

## Conclusions

Bioreactors fill a critical niche in the commercialization and clinical translation of cell-based therapies and drug-testing platforms. Current trends suggest an increased emphasis on manufacturing needs. This includes scaling up of suspension culture bioreactors to industrial sizes and modifications of tissue engineering bioreactors to enable the formation of patient-specific grafts that are of therapeutically relevant sizes. In spite of the many scientific and technical advantages of these systems, regulatory requirements may prove to be significant barriers to their clinical application. For lab-on-a-chip systems, major advancements in monitoring, control, and fabrication techniques are resulting in progressively more complex systems that more closely mimic human physiology and capture the interactions of multiple organs. The establishment of low-cost platforms will have a significant beneficial impact on the future of disease modeling and drug testing.
